# The Role of Religious Involvement in the Relationship Between Early Trauma and Health Outcomes Among Adult Survivors

**DOI:** 10.1007/s40653-015-0067-7

**Published:** 2015-11-23

**Authors:** Katia G. Reinert, Jacquelyn C. Campbell, Karen Bandeen-Roche, Jerry W. Lee, Sarah Szanton

**Affiliations:** 1 Johns Hopkins University School of Nursing, Baltimore, MD USA; 2 Johns Hopkins Bloomberg School of Public Health, Baltimore, MD USA; 3 Loma Linda University School of Public Health, Loma Linda, CA USA

**Keywords:** Abuse, Religious coping, Child abuse, Forgiveness, Gratitude, Family violence

## Abstract

The purpose of this study was to determine the role of religious involvement and related indicators - religious coping, intrinsic religiosity, forgiveness and gratitude - in reducing the negative impact of early traumatic stress on the mental and physical health of adult survivors. Multiple linear regressions were used to analyze self-reported data of 10,283 Seventh-day Adventist men and women across North America. The study also included an original analysis on a subsample (*n* = 496) of the larger group, examining diabetes risk factors in conjunction with Adverse Childhood Events (ACE) data. Higher early trauma scores were associated with decreased mental health (*B* = −1.93 *p* < .0001) and physical health (*B* = −1.53, *p* < .0001). The negative effect of early trauma on mental health was reduced by intrinsic religiosity (*B* = .52, *p* = .011), positive religious coping (*B* = .61, *p* = .025), forgiveness (*B* = .32 *p* = .025), and gratitude (*B* = .87 *p* = .001). Adult survivors of early trauma experienced worse mental and physical health; however, forgiveness, gratitude, positive religious coping, and intrinsic religiosity were protective against poor mental health. The findings support a holistic perspective in the care of childhood trauma survivors.

## Background

Early traumatic stress (ETS) has been documented as a predictor of adult negative mental and physical health (J. Campbell et al. 2002; Felitti et al. 1998). These adverse childhood experiences (ACE) include child abuse, exposure to parental intimate partner violence (IPV), and other types of family dysfunction which have been linked in a dose–response fashion to a range of adverse health outcomes which include: substance abuse, post-traumatic stress disorder, depression, cardiovascular disease, cancer, and premature mortality as well as adult perpetration of and victimization from family violence (D. W. Brown et al. 2009; CDC 2010; Cook et al. 2011; Felitti et al. 1998). Recently, researchers have also found associations between ETS and higher Body Mass Index (BMI), obesity, and type 2 diabetic risk (Rich-Edwards et al. 2010; Wegman and Stetler 2009). Although epidemiological links are strong, the mechanisms underlying these relationships have not been well delineated.

Not all people exposed to early traumatic experiences develop negative mental and physical health outcomes (Ai and Park 2005; Feder et al. 2009). Coping strategies play an important role in this phenomenon, and a number of studies suggest that religious involvement may be an important source for coping with trauma (Gillum et al. 2006; Pargament 1999; Southwick et al. 2005). However, many of the studies that have examined the role of religious involvement have conceptual and design flaws. This study addresses these flaws while examining the influence of religious involvement on the relationship of ETS with adult mental and physical health in a large multi-racial sample of Seventh-day Adventists (*N* = 10,253).

### Religious Involvement as a Protective Factor

Many community, family, and physiological tactics are used as coping strategies and protective factors that facilitate resilience in studies of general stressors and these include cultivating positive emotions such as gratitude, forgiveness (Giacomo and McCullough 2006; Krause and Ellison 2003), and religion/spirituality involvement (Pargament 1999). Religious involvement (RI) plays a significant role in many lives and has been defined as an institutional affiliation, beliefs, practices, or adopted behaviors that are guided by a religious denomination or community of faith (Reinert and Koenig 2013). Based on a meta-analysis of the last 10 years of research on religion and health, H. G. Koenig et al. (2012) reported that RI was significantly (*p* < .05) protective against emotional disorders (less depression, suicide, substance abuse) and positively associated with positive emotions/virtues (better well-being, happiness, meaning, purpose, hope, forgiveness, gratitude, altruism, compassion). Religious involvement was also significantly related, although less strongly, to physical health in the areas of heart disease, hypertension, cerebrovascular disease, dementia, immune function, endocrine function, cancer, and overall mortality. Lastly, the authors also found positive associations between RI and healthy behaviors such as exercise, less tobacco use, better diet, and greater likelihood of participating in disease screening behaviors with 52–90 % of studies on a given health behavior having significant (*p* < .05) associations. In some cases, these RI variables acted as mediators of health indicators (H. G. Koenig et al. 2012). Many of the above studies had small samples (Ahrens et al. 2010; Pargament 1999; Schneider and Feltey 2009), poorly explicated conceptual and operational definitions of RI (Reinert and Koenig 2013), inadequate representation of ethnic minorities, males and older age groups, sole reliance on self-report methods, and confounding of socioeconomic status (SES), race/ethnicity, and education (Wegman and Stetler 2009).

### Early Trauma, Religious Involvement, and Health Outcomes Summary

While a review of violence research over the last two decades identified some evidence of religious involvement (RI) attenuating the negative effect of trauma on mental health, only a limited number of studies in English were found to include the intersection of ETS, RI, and mental health outcomes, with none including physical health outcomes. Using child abuse as a primary keyword followed by adult survivors of child abuse and religion, religion and psychology, spirituality and mental health, and religious coping, four studies were identified in the Cumulative Index to Nursing and Allied Health Literature (CINHAL) and Medline databases measuring mental health outcomes, such as suicidality (Dervic et al. 2006), depression and anxiety (Gall 2006), PTSD, eating disorders (Krejci et al. 2004), hope, self-acceptance, personal growth (Gall et al. 2007), and factors facilitating effective transition from a traumatic past experience (Hall 2003b). Instruments used to measure RI and spirituality were confounded with mental health (e.g., questions related to existential well-being), putting into question some of the findings (Dervic et al. 2006; Krejci et al. 2004). In a qualitative study (*N* = 55), Hall (2003a) found that spiritual connection was one of six factors facilitating a healthy transition from past traumatic experiences among mostly African American women ETS survivors, but little information was given as to the analytic procedures. Similarly, one cross-sectional study of 101 men and women survivors of childhood sexual and physical abuse, found that those who highly valued their spiritual experience had better transitions from trauma, when compared to those with high levels of spiritual discontent (Gall 2006; Gall et al. 2007). The researchers reported that negative religious coping (e.g., being angry at God for the abuse) predicted greater levels of depressive mood, compared to those reporting positive religious coping, forgiveness, and a positive connection with God. Furthermore, religious coping seems to be protective of additional negative mental health symptoms, such as suicidal ideation, on those with depression. Dervic et al. (2006) found that among 119 depressed inpatients, those with a history of child abuse who highly valued religious beliefs and had moral objections to suicide reported experiencing less suicidality and an increased benefit from treatment if they were encouraged to pursue those values (Gall et al. 2007).

Most of the people in these studies experienced a history of child abuse but the type, exact age, and duration of the ETS was not well identified in every case. No studies evaluated neglect, psychological abuse, or witnessed violence. These studies had limitations related to small convenience samples (largest sample had 119 people), inadequate gender and ethnic representation, (mostly women, and/or one racial group) lack of control groups, and confounding of some instruments used to measure RI or spirituality (Bent-Goodley 2005; Dervic et al. 2006; Gall 2006; Gall et al. 2007; Hall 2003b; Krejci et al. 2004). These limitations were addressed in our study, which included a larger sample of men and women with racial diversity from across North America, including participants with and without a history of ETS that included all types of child abuse in addition to neglect and those who witnessed parental abuse.

### Philosophical Framework

**Fig. 1:**
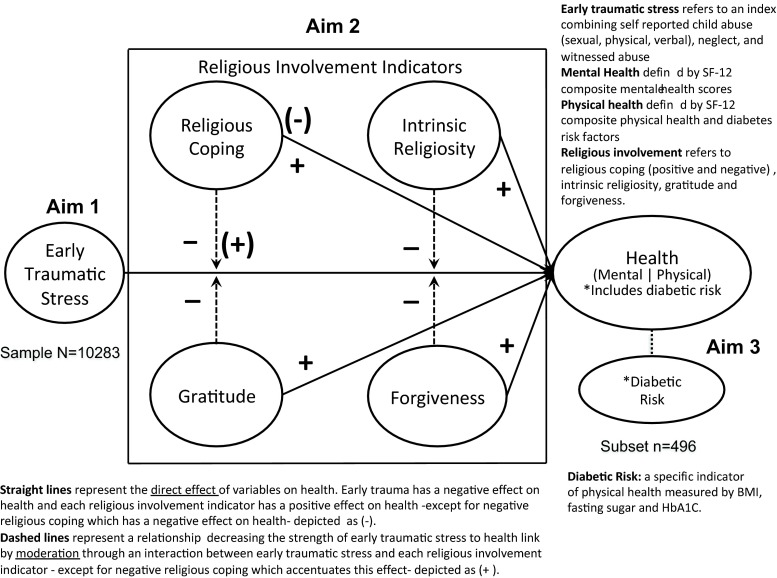
Proposed trauma-religious involvement-health model. Note: Aim 1–3 incorporates the society-to-cells resilience framework

This study was guided by the Society-to-Cell Resilience Framework (Szanton and Gill 2010) – a model that conceptualizes individual, society, and community factors as having an effect at the cellular level influencing genetic expression (epigenetics). The Society-to-Cell framework proposes that a number of protective factors (e.g., society, family, individual, physiological) may strengthen resilience in individuals, which in turn influences their stress response (Szanton and Gill 2010). This buffering of the negative impact of ETS as a severe stressor could allow ETS survivors to experience better health outcomes (Southwick et al. 2005). This framework has been cited by over a dozen publications as a valid model of resilience. As such, it was chosen to inform the development of the ETS-RI-Health model (Fig. 1) tested in this study through aims 1–3 below. In the ETS-RI-Health model, ETS has a direct association with mental and physical health (includes diabetic risk of a subsample). Furthermore, the RI indicators of religious coping and intrinsic religiosity, as well as gratitude and forgiveness, have a positive association with health as well as a moderating effect on the ETS-health relationship.

## Methods and Design

### Research Aims and Design

The study aims were to: (a) Determine the relationship of early traumatic stress (ETS) with adult mental and physical health; (b) Determine the influence of religious involvement (RI) on the relationship of ETS with adult health by testing the moderating effects of religious coping (positive and negative), intrinsic religiosity, gratitude, and forgiveness on the above relationship; (c) Examine whether or not ETS is associated with adult diabetic risk as measured by HbA1C, fasting glucose, and obesity (BMI) (subsample *n* = 496).

The study used a cross-sectional design to test the ETS-RI-Health model by analyzing data collected from a sample of two NIH-funded epidemiologic studies: the larger Adventist Health Study-2 (AHS-2-R01CA094594) (T. L. Butler et al. 2008) and the Biopsychosocial Religion and Health Study (BRHS-1R01AG02634) (J. W. Lee et al. 2009), which was randomly selected from AHS–2. The study also includes a first original analysis on a subsample of the BRHS data (*n* = 496), examining diabetes in conjunction with ACE data. The AHS-2 and BRHS explores how religious beliefs and practices may affect both cause-specific mortality and quality of life, and have not previously analyzed ETS as described nor the RI indicators of this study.

### Sample Description

The Seventh-day Adventist Church, whose members comprise 1.2 million members in the US, is a conservative Protestant denomination that has extensive longitudinal studies on health outcomes and longevity funded by the NIH. Adventists hold a religious doctrine that promotes health and disease prevention by advocating temperate lifestyles, such as regular physical activity, a vegetarian diet, and the avoidance of tobacco and alcohol - Belief #22 of 28 - (http://www.adventist.org/beliefs/living/christian-behavior.). These lifestyle practices have long been seen as the probable reason for lowered disease incidence and increased longevity in Adventists (G. E. Fraser 2003). There is less difference in SES in Black and White Adventists than in the general population, resulting in less of the usual confounding of race and SES than is common in health research (Table 1).

**Table 1:** Demographic characteristics of sample

VARIABLE, n (%)	MAIN SAMPLE (*N* = 10283)	SUBSAMPLE (*N* = 496)
Age, mean (Years)	61.7^a b^	69 ^c d^
Gender		
Male	3333 (32)	181 (37)
Female	6946 (68)	312 (63)
Race		
Blacks	3750 (36)	192 (41)
Whites	6533 (64)	281 (59)
Education		
≤ High or trade	2441 (24)	32 (7)
Some College	2237 (22)	91 (19)
College degree	3403 (33)	160 (34)
≥ Graduate School	2085 (21)	185 (40)
Income (Self) *mean* (past 12 months)	$40 K (4.4)^e^	$31 K (4.0)^f^
< $10 K (1)	5353 (17)	44 (10)
$11–20 K (2)	1987 (20)	56 (13)
$21–30 K (3)	1660 (17)	63 (14)
$31–50 K (4)	2159 (22)	100 (22)
$51–75 K (5)	1233 (13)	93 (21)
$76–100 K (6)	532 (5)	49 (11)
> $101 K (7)	508 (5)	43 (9)

^*a*^ *Range* = 30–106; ^*b*^ *Range* = 36–102 ^*c*,*d*^ *Range* = 36–102 ^*e*,,*f*^ *SD* 2

### Sample Recruitment and Data Collection

Original recruitment for the AHS-2 was done in Seventh-day Adventist churches in the US and Canada. About 96,000 church members consented to enroll in the study. For the BRHS study recruitment, questionnaires were sent to 20,000 randomly selected participants from the AHS-2 and 11,000 consented to participate by filling them out and returning them during 2006–2007. Among those who responded, 34 % self-identified as Black, 60 % as White, and 6 % as other. Ages of the participants ranged from 30 to 106 years (M = 62, SD = 14) and 54 % had college education (Table 1). Next, 496 (55 % White, 45 % Black) of the nearly 11000 lived near the Loma Linda University Medical Center and consented to have blood draws and biomarker data collected at this site. This study includes Black and White participants in the model testing, reducing the sample to *N* = 10,283.

### Measures

Early traumatic stress (ETS) was operationalized as exposure to one or more types of child abuse (physical, psychological, sexual), neglect, and witness abuse. Items were taken from a modified version of the Trauma Inventory Scale (Cusack et al. 2004; McHugo et al. 2005) (α = .73), Ryff’s Relationship Scale (Ryff et al. 2004) (α = .81–.84), and the Risky Family scale (Taylor et al. 2006) (α = .85) adapted from Felitti’s ACE measures. The instrument asks about five types of ETS: (a) witnessed family violence – one question (e.g., how often a parent behaved violently towards the other parent), (b) neglect - one question (e.g., how often felt neglected), (c) psychological abuse – two questions (e.g., how much parents insulted, swore at, ignored), (d) physical assault – four questions (e.g., how much parents pushed, kicked, slapped), (e) sexual assault– two questions (e.g., had sexual contact with anyone 5 years older before age 13; anyone used pressure or threats to have sexual contact before age 18). A cutoff of 2 (e.g., “*a little*”) on a 4-point Likert or 5-point Likert scale (e.g., *once in a while*) was used to identify exposure to each type of ETS. Early Traumatic Stress (any type) was built into a binary variable (0 = no exposure to any type of ETS as noted above; 1 = exposure to at least one type). Some analyses were done using ETS (all types) for 1 = exposure to all five types of ETS for comparison with ETS (any type). In terms of validity, all items in the ETS measure were examined by experts in the field and were determined to match the content of many ACE measures, providing face and content validity. Then, an eigenvalue analysis done using principal components was strongly supportive that the ETS items measure a uni-dimensional construct with one strongest factor noted. These steps formed the validity basis for an overall measure of ETS covering the five distinct types of childhood traumatic experiences used here.

Religious involvement factors included intrinsic religiosity, religious coping (positive and negative), gratitude, and forgiveness. Items came from: the Duke University Religion Scale (DUREL) (H. G. Koenig et al. 1997) - three items assessing intrinsic religiosity (α = .72- α = .83) with questions related to private prayer, study, or church attendance; the Brief RCOPE scale (α = .73 (Pargament 1999) - 10 items assessing positive or negative use of religion as a coping mechanism which has two subscales (positive religious coping, five items - e.g., I tried to make sense of the situation with God’s help) and negative religious coping (five items - e.g., felt punished by God); The Gratitude scale (McCullough et al. 2002) (α = .61)- six questions such as “I have so much in life to be thankful for;” Idler (1999) (α = .58) forgiveness scale with one question, “I forgiven those who hurt me.”

Mental and Physical health was measured using the SF-12 version 2 which is derived from the SF-36 (Ware et al. 2002) (α = .84–.95). The SF-12 measures mental and physical health related to quality of life through a mental component summary (MCS) and a physical component summary (PCS) calculated from the 12 questions. These were used as our mental health and physical health outcome variables. In addition, in the smaller clinical sample, diabetes risk factors of fasting sugar (mg/dL) and hemoglobin A1c (%) were drawn by a certified phlebotomist using a standard protocol, while body mass index (BMI) was calculated based on objective measures of height (cm) and weight (Kg).

Permission to use the de-identified data was received from Loma Linda University and the parent study principal investigator. The study received approval from the Internal Review Board at the Johns Hopkins University.

## Data Analysis

Data analysis was performed using STATA version 12 (Hamilton 2009). Initial analysis was exploratory to identify outliers, evaluate assumptions, and identify multi-collinearity that could preclude mutual inclusion of variables in any of the multivariate analyses. Multiple linear regression (Dupont 2009) was used to test the theoretical model. The independent variable of ETS was created using a binary measure for the presence or absence of abuse (physical, psychological, sexual), neglect, and witnessed domestic abuse. The dependent variables were mental and physical health scores (measured by SF12). On the subsample (*n* = 496) the dependent variables were BMI, fasting glucose, and HbA1c (diabetic risk factors). Moderators were the RI variables of intrinsic religiosity (IR, scores 1–7), religious coping (RC, scores 1–5), gratitude (scores 1–7), and forgiveness (scores 1–7). Moderators were tested according to Barron and Kenny (1986), and Judd et al. (2001) using regression procedures. Descriptive statistics were calculated for all study variables noted above. Data was summarized with frequencies and groups were compared using chi-square or *t*-tests as appropriate for each variable. Analysis was adjusted for potential confounders of age, gender, and race (Blacks and Whites) based on the descriptive statistic results and since previous studies have documented differences in mental and physical health outcomes related to these in the setting of RI variables (H. G. Koenig 2008, p. 29). The primary outcome for aims 1–2 was adult health (mental and physical), and for aim 3 it was diabetic risk. A power calculation was done in order to find how many standard deviations of the outcome (K SD) were detectable (Rosner 2010). Power was reported as standard deviations in health outcome per unit increase in trauma score. For Aim 1: for power = 0.80 the effect size was K = 0.03 (SD = 0.678, *N* = 10283) and for power = 0.90 the detectable effect size was k = 0.046; for Aim 2: the detectable effect size for a power = 0.80 was K = 0.08, and when power = 0.90 the effect size was K = 0.09 (*N* = 10,283 SD = 0.678). Thus, the study design provided ample power to detect even a subtle effect for the 2-way interactions. Multiple imputation was conducted to account for missing data (Schafer and Graham 2002).

## Results

### Demographic Characteristics

Among the larger sample (*N* = 10,283), the *mean* age was 61.7 years (*SD* 14) with a range of 30–106, 32 % were male and 68 % female, with Blacks comprising 36 %, and Whites 64 % of the sample. About 11 % self-reported having had a diagnosis of diabetes, 25 % having a BMI >30 and the *mean* BMI was 27.2 (*SD* 6.1). Over half of the group reported having a regular exercise program with 45 % engaging in vigorous activities at least three times per week. Nearly half were not employed due to retirement and *mean* income was $40 K (SD 25 K-lower, 36 K-upper) (Table 1). In terms of associations, the prevalence of ETS in the sample (those experiencing any physical, emotional, sexual, neglect, or witnessed before age 18) was 67 %, whereas 9 % reported having at least three ETS types combined and 5 % experienced all five types combined. Among those, the prevalence of ETS among women was significantly higher than among men (69 versus 65 %) and three times as many women reported having had all types of ETS (6 % females versus 2 % males). As far as health outcomes, the *mean* mental health score in the sample was 53 (*SD* 9) and for physical health the *mean* was 48 (*SD* 11).

Demographics for the subsample were similar to overall sample, except there were higher percentages of Blacks (41 versus 36 %), less Whites (59 versus 64 %), slightly more males (37 versus 32 %) and less females (63 versus 68 %) when compared to the overall sample (Table 1). Early Traumatic Stress prevalence was comparable with the larger sample although slightly lower for any type of abuse (62 versus 67 % in the overall sample) as well as for each of the ETS subscales.

In terms of diabetic risk factors, the objective *mean* BMI was the same as the larger sample (27.4, *SD* = 5.7) with a slightly older sample of *mean* age 69 (*SD* 12). The reported diabetes diagnosis in the subsample was 12 % (nearly the same as the 11 % in the larger sample), with the prevalence of objective diabetes measures of HbA1c >6.5 (11 % *mean* = 5.9 *SD* = 0.7) and fasting sugar >120 (7 % *mean* = 94 *SD* = 21) being lower than in the general population (13.7 % among ages 45–64 or 26.9 % ages > 65) (CDC 2012). About 74 % of the subsample had a college degree or higher level of education compared to 54 % of the larger sample (Table 1).

### Early Traumatic Stress, Religious Involvement, and Health

Ninety per cent of participants scored the highest possible points (7) on levels of religious involvement, whereas 90 % of participants scored on the low end of the RC for negative RC scale (2 or lower). Results of our analysis for the overall sample indicate that higher ETS scores were associated with a significant reduction in mental health (*B* = −1.93 *p* < .0001) and physical health (*B* = −1.53 *p* < .0001). These results show the unstandardized coefficient B where a history of exposure to at least one type of ETS or more was associated with a mean reduction in mental health of −1.93 and a mean reduction in physical health of −1.53 (both on a raw scale of 0–100). When we analyzed this link among those with a history of ETS exposure to all five types of abuse (*n* = 447), we noted that the mean reduction in mental health (B = −2.80, *p* = <.0001) was larger than among those with one or more ETS exposures. Likewise, for those exposed to all five types of ETS, the mean reduction in physical health was −3.20 (*p* < .0001). Overall, those with higher exposures to ETS subtypes had nearly 1 point greater reduction in mental health (−2.80) and more than twice as much a reduction for physical health (−3.20).

However, this adverse effect on health was moderated by some of the RI indicators in the model. As hypothesized, upon adding two-way interactions of ETS*RI variables, the negative effect of ETS on mental health was reduced by positive religious coping (*B* = .61 *p* = .025), intrinsic religiosity (*B* = .52, *p* < 0.011), forgiveness (*B* = .32, *p* = .025), and gratitude (*B* = .87, *p* = .001) (Table 2). The protective effect of forgiveness and gratitude was greater among those in the ETS group who experienced all five types of traumatic experiences (*B* = .85 *p* = .003 for forgiveness, and *B* = 1.30 *p* = .011 for gratitude). These unstandardized *B* coefficients reflect a small but robust statistically significant reduction on a 0–100 point mental health scale. Negative religious coping had a negative impact by -.05 points on the mental health scale for those with history of ETS but this effect was not statistically significant (*p* = .900) (Table 2). Contrary to our prediction, the negative effect of ETS on physical health was not reduced by the RI factors. Finally, high levels of ETS in the biomarker subsample (*n* = 496) were not associated with significant increases in diabetic risk factors of fasting sugar, hemoglobin A1C, and BMI (although a significant positive association of ETS with self-reported BMI was noted post hoc analysis of the overall sample, *p* < .0001). However, in line with previous studies, Whites had a lower fasting sugar by 4 mg/dL than Blacks (*p* < .05); women had a lower fasting sugar than men by 5 mg/dL (*p* < .05); and for every 1 year increase in age, fasting sugar increased by .28 md/dL (*p* < .01). Similarly, for HbA1c, Whites had a .32 % lower HbA1C than Blacks (p < .0001) and for each 1 year increase in age HbA1c increased by .01 % (*p* < .05). Although the analysis on the objective measures of diabetes risk did not show a link with ETS, post hoc analysis by type of ETS demonstrated a positive association between sexual abuse and fasting sugar (B = 6.87, *p* = .013, CI 1.47, 12.27), controlling for age, gender, and race. The analysis remained significant after adding additional controls for education and income (B = 5.82, *p* = .040, CI .26, 11.38).

**Table 2:** Multiple regression: moderation analysis of religious involvement

	B	SE	95 % CI
MENTAL HEALTH			
ETS X Intrinsic Religiosity	.52**	.21	[.12, .93]
ETS X Positive Religious Coping	.61*	.27	[.08, 1.13]
ETS X Negative Religious Coping	−.05	.42	[−.88, .77]
ETS X Forgiveness	.32*	.14	[.04, .60]
ETS X Gratitude	.87**	.26	[.36, 1.38]
PHYSICAL HEALTH			
ETS X Intrinsic Religiosity	.01	.25	[−.49, .48]
ETS X Positive Religious Coping	−.15	.32	[−.78, .49]
ETS X Negative Religious Coping	−.18	.51	[−1.17, .81]
ETS X Forgiveness	.33	.17	[−.01, .67]
ETS X Gratitude	.06	.33	[−.58, .70]

Multiple imputation data used to account for missing variables of mental and physical health *B* = Unstandardized interaction coefficients. Statistically controlled for, age, gender, race

*p < .05 **p < .01 ***p < .0001

## Discussion

As hypothesized, adult survivors with highest levels of ETS reported worse mental and physical health when controlling for age, gender, and race, thus affirming the direct link of ETS on health in our ETS-RI-Health model. The results are in line with other studies reporting the negative impact of ETS on health (CDC 2010; Felitti et al. 1998; Springer et al. 2007). It is important to note that unlike the ACE studies, these results show a link of exposure to as few as one type of abuse whereas in the ACE study led by Felitti and colleagues linking adverse childhood experiences to adverse health in adult survivors, the higher prevalence of disease was seen primarily with exposure to three or four types of ACE (odds ratios from 1.2 to 3.9 depending on the condition), whereas exposures to one or two types of ACE ranged from 0.7 to 1.0 in some cases (no increased disease risk) (Felitti et al. 1998). Even though we did not measure specific conditions as the ACE study did, overall mental and physical health is significantly affected in a negative way even by a low exposure to ETS. The prevalence of ETS by type of child abuse in this study is higher than the prevalence in the ACE study (38 versus 10.8 % for physical, 38 versus 11.1 % for emotional, 23 versus 22 % for sexual, 28 versus 12.5 % witnessed) (Felitti et al. 1998). Likewise, the prevalence of overall ETS (any child abuse, neglect, or witnessed abuse) was also higher than in the ACE study (67 % compared to 52 % for ETS ≥1 reported).

The significantly higher ETS prevalence among women (69 versus 65 % in men) and among Blacks (75 versus 64 % in Whites) is in line with ACE data (54 % in women and 46 % in men; 61 % in Blacks versus 50 % in Whites) and more recent CDC data analyzing ACE in five states (2010) (61 % versus 58 % in men, 63 % versus 58 % in Whites) (CDC 2010). It is important to note that the ACE studies do not include the neglect measure included in this ETS measure, and that could help explain the higher overall prevalence in our study. However, ACE studies also have two measures of household dysfunction (household member having mental illness and/or and criminal behavior/imprisonment) that are not included our ETS measure. The higher ETS prevalence among women and Blacks in this study is also consistent with some CDC data (2010) where adult females reported a prevalence of sexual abuse over twice as much (17.2 vs. 6.7 %). The overall ETS prevalence in the biomarkers subsample (*n* = 496) was lower than reported in the study of the diabetic women survivors (62 vs. 65 % in the women’s study) (Rich-Edwards et al. 2010). Similarly, the prevalence of physical child abuse was higher in the women’s study sample than in this sample (54 vs. 34 % for this total sample, 26 % for those with diabetes diagnosis only, and 20 % for females with diabetes diagnosis).

As predicted, the examination of the role of RI in this gender inclusive sample demonstrated that intrinsic religiosity, positive religious coping, and positive virtues of forgiveness and gratitude seem to be important factors in reducing the negative impact of ETS on mental health (Table 2), as our ETS-RI-Health model suggests (Fig. 1). While this finding is consistent with a few small studies that have examined this question among mostly female samples (Gillum et al. 2006; Silva et al. 1997; Springer et al. 2007), the results here suggest a protective benefit for both genders and in a much larger sample. However, studies suggest there are gender differences in regards to religious involvement factors (e.g., women have higher levels of religious attendance than men) (H. G. Koenig 2008), thus further investigation of gender differences in regards to the protective effect of these religious coping strategies could shed further light on how religious involvement indicators might impact men and women separately. The same is true of race. The benefit for mental health is seen here in a sample with both Blacks and Whites. However, historically, Blacks report seeking religious coping strategies more readily than their White counterparts (Bent-Goodley 2005). Thus, investigating racial differences could be helpful in shedding further light on how these may impact Blacks and Whites differently.

While high levels of negative religious coping (RC) were significantly associated with worse mental health (*B* = −4.73 *p* < .0001), it did not significantly exacerbate the association of ETS with worse mental health (*B* = −.05 *p* = .900) as we had hypothesized. This is probably related to the fact that the use of negative RC in this sample was quite low (*mean* 1.42, *SD* .51, *range* 1–5). The standard deviation (SD) for all the positive RI variables is wider than the SD for the negative RC noted above. This could have contributed to a lack of variance and help explain the small effect observed. It is also possible that social desirability in this sample could have affected the way participants answered questions about their use of negative RC (e.g., blaming God may be perceived as an undesirable behavior, even though they may feel that way). This could have masked the real use of negative RC in the sample, however it seems that survivors in this sample overwhelmingly chose to use positive RC (*mean* = 4.2, *SD* .71, *range* 1–5), forgiveness (*mean* 6.18, *SD* .95, *range* 1–7), and gratitude (*mean* 6.31, *SD* .75, *range* 1.3–7) than negative RC (Table 2). This low use of negative RC, even among ETS survivors, coincides with findings from Gall (2006) where men and women survivors of child abuse seemed to lean on positive religious coping methods like forgiveness, and less negative forms of religious coping, such as blaming God for their abuse (Hall 2003a).

Paths for both gratitude and forgiveness were supported in our analysis (Fig. 1). As hypothesized, gratitude acted as a moderator of the ETS- mental health effect, with even higher benefit for those in the ETS group who experienced all types of abuse. The increase in mental health was nearly over 50 % greater for them when compared to others in ETS with 1–4 types of exposure. Gratitude is a positive virtue that is reinforced and encouraged by various religious faiths (Giacomo and McCullough 2006), although not usually considered as an indicator of religiosity, and while studies have examined gratitude benefits on health (Giacomo and McCullough 2006; Krause 2009) no studies have studied this virtue in the setting of ETS exposure. The findings here suggest further studies should be done using a gratitude scale with higher reliability to examine gratitude as a potential effective intervention to improve mental health outcomes among survivors.

With regards to forgiveness – another positive virtue strongly encouraged by but not unique to religious doctrines – a potentially protective effect for mental health was also noted as hypothesized. This mental health protection was nearly three times greater for those in the ETS group exposed to all types of trauma. This moderation of the ETS-mental health relationship by forgiveness is consistent with the general literature on forgiveness as noted earlier (Giacomo and McCullough 2006; Krause and Ellison 2003). However, no large quantitative studies examined forgiveness of the perpetrator specifically. We used one forgiveness question that specifically measured how often the participant forgave those that had hurt them in the past. This question was an approximation of measuring forgiveness of the perpetrator of abuse. On a test-retest reliability over 3 years time this forgiveness measure had adequate correlation (.45). Based on this, the potentially protective effect of forgiveness found in our analysis may suggest a benefit for mental health among those in the sample who had the highest levels of forgiveness towards others, possibly including perpetrators of abuse. Future studies should evaluate this question more specifically, and perhaps other types of forgiveness. Also, it is important to note that while forgiveness and gratitude are encouraged and embraced as a duty in many religions or communities of faith, people who do not subscribe to a religion may also embrace these virtues. Therefore, further studies should be conducted among non-religious samples to evaluate whether the buffering noted here may be replicated.

With regards to the ETS-RI-health model paths related to physical health, there was no strong evidence that the RI indicators moderated the negative effect of ETS on health in the large sample, contrary to our prediction. Among the four RI indicators, none reached statistical significance, although forgiveness had near significant results (*B* = .32, *p* = .051). Likewise, in terms of an objective measure of physical health, the exploratory analysis done on the subsample (*n* = 496) to examine the link of ETS to diabetic risk factors (aim 3) did not find any significant association either, even though other studies in larger samples have shown that link. The lack of support for the physical health path in the ETS-RI-Health model may be explained. Although the literature notes links of the RI indicators to positive physical health, these links are in many cases mediated either by mental health or lifestyle factors (H. G. Koenig et al. 2012). The overall good health, prevalence of healthy behaviors (physical activity, healthy diet), and low prevalence of adverse health behaviors (such as smoking, alcohol, or drug use) in the Adventist population is a very likely explanation of why we did not see RI moderation for physical health. The pathways on which ETS-physical health might otherwise have acted may not be operant in a Seventh-day Adventist group due to the protective health behaviors.

With regards to the subsample, the lack of association of high levels of ETS and diabetes risk factors that was found in other large epidemiological studies showing this link (CDC 2010; Rich-Edwards et al. 2010) may be partially explained by differences in the ETS measure definition and sample characteristics. In the ACE study, Felitti et al. (1998) found that only those with exposure to three or more ACE categories had a higher odds ratio for diabetes (*OR* = 1.2 for those with three ACE categories, and *OR* = 1.6 for four categories). Those in their study having 0–2 ACE categories had OD = 0.9–1.0 (no increased risk). Since ETS in the current study is binary and a presence of ETS is defined as experiencing one or more ACE categories of child abuse, the results do not contradict the ACE study findings - the majority of the ETS sample (91 %) in this study experienced 1–2 ACE categories. In addition, the ACE study sample size was 9508 whereas our sample evaluating diabetes was much smaller (*N* = 496).

In terms of differences in sample characteristics, the overall diabetes prevalence (11 % - self report, 11 % by HbA1c levels, 7 % by fasting sugar levels) was lower on the subsample than the national levels (26.9 % of the general population >65 years old - CDC’s 2010 National Health and Nutrition Examination Survey) (CDC 2012). The low prevalence of the diabetic risk factors in this smaller subsample (*n* = 496) may have yielded insufficient variance or power to detect differences. The levels of fasting sugar (*mean* =94; *SD* = 21) and HbA1c (*mean* =5.9; *SD* = 0.7) for most in the sample (*mean* age 69) were not indicative of diabetes. Of the 35 people (7 %) who had a fasting glucose indicative of diabetes (>120) in the subsample, 22 (63 %) had a history of ETS – almost exactly the same as the overall prevalence in the subsample (62 %), so no evidence of differences between the groups. In the case of HbA1c, 56 (11 %) people in the sample had a value >6.5, which is indicative of diabetes, and 27 (49 %) of them had a history of ETS. Thus, the number of diabetics in our small subsample (*n* = 496) compared to the larger epidemiological samples that studied this ETS-diabetes link (*N* = 8056, *N* = 67853) (Felitti et al. 1998; Rich-Edwards et al. 2010) may have contributed to a lack of variance and power to detect differences among ETS groups.

Also, when we compare this study’s results to the larger sample of women in the nurse’s study, the prevalence of physical abuse was much higher there than in this subsample [54 % there versus 20 % (women only) 26 % (men/women) with diabetes diagnosis in our subsample – overall prevalence of physical abuse among those with and without diabetes was 34 % (Rich-Edwards et al. 2010). Since they reported dose–response association of physical abuse and diabetic risk, the lower prevalence of physical abuse in this subsample could perhaps help explain the lack of association of ETS to diabetic risk. Also, since in this study links were found between specific types of abuse to diabetes, we performed a post hoc analysis of specific types of ETS and found a strong significant association between sexual abuse and increased fasting sugar (B = 6.9, *p* = .013, CI 1.5, 12.3) controlling for physical activity, diet, age, gender, and race. This association remained significant when we added income and education to the model (B = 5.8, *p* = .040, CI, .25, 11.38).

Finally, another lifestyle difference between this subsample and the ACE studies is the lack of smoking (.8 % among those with ETS in our study) and alcohol abuse (<10 % reported using it), which may have contributed significantly to a positive effect on the physical health of survivors in this study. Felitti and colleagues found that ETS was significantly associated with smoking, in contrast to the population in our study. The prevalence of health behaviors in the ACE study ranged from 8 to 16 % for smoking depending on ETS exposure, and 6–16 % considered themselves alcoholics (Felitti et al. 1998). In addition, Rich-Edwards et al. (2010) found in the nurse’s study that alcohol and BMI weakened the association of abuse to diabetes among the women studied, and physical inactivity was low overall in our sample. Although researchers attributed this reduction in risk mostly to BMI, further examination should be done to evaluate gender differences as to how smoking and other lifestyle factors such as diet, alcohol use, and physical activity may impact the physical health and diabetic risk of ETS survivors.

## Study Strengths

This is the first study to analyze religious involvement variables and the related positive virtues of forgiveness and gratitude in a large sample of adult survivors with health outcome data. Also, having measures for spirituality/religion that do not confound with mental health outcomes brings more credibility to its moderating effect on the relationship of ETS and mental health. Additionally, the inclusion of males and females, as well as a high percentage of Blacks and Whites with higher income levels and education than in most previous studies of violence allows us to reduce SES related confounding. Also, being able to compare adults with and without a history of ETS provides more accurate results in terms of the moderation of RI factors for health outcomes. Lastly, examining the specific aims of this study in relation to religious involvement in a single denomination eliminates the need to control for differences in beliefs since they have a shared religious tradition.

## Limitations

This study has a number of limitations. First, since the study uses data from a larger NIH-funded study, the available data for this secondary analysis was somewhat limited. The measures of abuse and RI were already established and two of them (forgiveness and gratitude) had less than ideal, although acceptable, Cronbach’s alphas suggesting a potential problem with the internal consistency of the measures. In the case of forgiveness, the three different types of forgiveness measured (by God, of self, and of others who hurt me) accounted for the low alpha (α = .58). We attempted to minimize this problem by choosing only one item from the forgiveness measure that was particularly relevant since it related to forgiving the perpetrator of abuse, but a one item proxy for a scale is usually problematic. The test-retest reliability (3 year) on that single item of forgiveness was adequate (*r* = 0.45). The lack of racial diversity (other than Blacks and Whites) is another limitation as we will not be able to generalize the finding to Adventists of other racial backgrounds. However, the high prevalence of Blacks has allowed us to generalize findings to both Blacks and Whites (Adventists), whereas other studies lacked good representation Blacks or Whites in the same study.

We also note that the varying age of the participants and the self-report nature of the data collection may bring recall bias of ETS, although most studies accept the individual’s report of victimization as close to reality and prevalence of child abuse in this sample is close to that of the general population. Self-selection bias and convenience sampling of the sub-sample might have excluded people with diabetic risk factors or diagnosis as the Loma Linda area is known as a “blue zone” (Buettner 2008), suggesting that the Adventists in that area generally adopt healthier lifestyles than the general population. Furthermore, the sample is of Adventists, thus generalizability of the findings to the general population is another serious limitation; however, participants were randomized from many regions of the United States and the findings can be contextualized to populations of other faith groups with similar beliefs and similar lifestyles across North America. The analysis on the diabetes subset was limited by a low power, however the magnitude of the ETS prevalence was small, consistent with no strong effect in the population. Lastly, causal inference is not possible with a cross-sectional design since temporality of measures could not be established; however, despite the above limitations, these results laid the theoretical grounding for future longitudinal causal model testing.

## Conclusion

Despite the limitations of this study, it indicates that there is a high prevalence of ETS among a large sample of Adventists despite their high level of education and high religious involvement and that these childhood traumas have a significant negative effect on overall physical and mental health as is true in general populations. Furthermore, it shows that religious involvement factors (spirituality/intrinsic religiosity, religious coping, forgiveness, and gratitude) can be effective coping mechanisms to improve mental health outcomes for survivors of ETS. These effects were seen in this racially diverse sample of men and women. However, further investigation of this seemingly protective effect by gender and race could shed further light on how coping using religious involvement may differ among males, females, Blacks, and Whites. Despite the lack of statistically significant findings in regards to the specific effect of ETS on physiological diabetes indicators, this study poses questions regarding the need to explore potential mechanisms related to healthy lifestyles (particularly diet, smoking, and physical activity behaviors) that may affect the ETS-Health relationship. The analysis done here lays the theoretical grounding for future longitudinal causal model testing to evaluate these questions. Although religious coping, intrinsic religiosity, forgiveness, and gratitude are difficult to implement as treatments, some small studies have examined the effectiveness of counseling interventions that support these coping mechanisms in people who valued them with some effectiveness. Further research may include potential counseling interventions that support these positive virtues as protective strategies for people exposed to ETS.

This study contributes to scientific knowledge as it builds on the foundation for holistic care of the person, advancing future research on the path to examine prevention strategies and more effective interventions particularly for survivors of similar faith communities, or for those who culturally find religious involvement factors to be a preferred mode of coping. Advancing such understanding in clinical practice can provide a model aimed at strengthening resilience and reducing negative mental health outcomes for trauma survivors.
